# Visual attention during pediatric resuscitation with feedback devices: a randomized simulation study

**DOI:** 10.1038/s41390-021-01653-w

**Published:** 2021-07-21

**Authors:** Michael Wagner, Peter Gröpel, Felix Eibensteiner, Lisa Kessler, Katharina Bibl, Isabel T. Gross, Angelika Berger, Francesco S. Cardona

**Affiliations:** 1 Divison of Neonatology, Pediatric Intensive Care and Neuropediatrics, Department of Pediatrics, Comprehensive Center for Pediatrics, Medical University of Vienna, Vienna, Austria; 2 Division of Sport Psychology, Department of Sport Science, University of Vienna, Vienna, Austria; 3 Department of Pediatrics, Section of Pediatric Emergency Medicine, Yale School of Medicine, New Haven, CT USA

## Abstract

**Background:**

The aim of this study was to investigate the effect of feedback devices on visual attention and the quality of pediatric resuscitation.

**Methods:**

This was a randomized cross-over simulation study at the Medical University of Vienna. Participants were students and neonatal providers performing four resuscitation scenarios with the support of feedback devices randomized. The primary outcome was the quality of resuscitation. Secondary outcomes were total dwell time (=total duration of visit time) on areas of interest and the workload of participants.

**Results:**

Forty participants were analyzed. Overall, chest compression (*P* < 0.001) and ventilation quality were significantly better (*P* = 0.002) when using a feedback device. Dwell time on the feedback device was 40.1% in the ventilation feedback condition and 48.7% in the chest compression feedback condition. In both conditions, participants significantly reduced attention from the infant’s chest and mask (72.9 vs. 32.6% and 21.9 vs. 12.7%). Participants’ subjective workload increased by 3.5% (*P* = 0.018) and 8% (*P* < 0.001) when provided with feedback during a 3-min chest compression and ventilation scenario, respectively.

**Conclusions:**

The quality of pediatric resuscitation significantly improved when using real-time feedback. However, attention shifted from the manikin and other equipment to the feedback device and subjective workload increased, respectively.

**Impact:**

- Cardiopulmonary resuscitation with feedback devices results in a higher quality of resuscitation and has the potential to lead to a better outcome for patients.
- Feedback devices consume attention from resuscitation providers.
- Feedback devices were associated with a shift of visual attention to the feedback devices and an increased workload of participants.
- Increased workload for providers and benefits for resuscitation quality need to be balanced for the best effect.

## Introduction

Around 2% of pediatric intensive care unit admissions will experience cardiac arrest.^1^ Successful high-quality cardiopulmonary resuscitation (CPR) of in-house arrest may lead to the discharge of up to half of these patients.^1–3^ Training in a simulation setting improves the quality of CPR.^4,5^ Focus is on adequate chest compressions (CCs) (depth, frequency, recoil) and ventilations (frequency, tidal volume, leak, pressure) as well as few interruptions (short hands-off time). This may be achieved with real-time feedback devices used during resuscitation, which may assist in achieving a better quality of resuscitation.^6–11^ These devices relay information on compression as well as ventilation quality.^12–16^ Even though feedback devices are increasingly being used in educational and clinical settings, the resuscitation guidelines by the European Resuscitation Council still argue against routine implementation^17^ as evidence is still not clear-cut. Previous studies using the same feedback devices as in our study [QCPR Baby (Laerdal, Stavanger, Norway) for CCs and Neo Training (Monivent AB, Gothenburg, Sweden) for ventilations] have shown an improvement of resuscitation and ventilation performance.^6,18,19^ However, these devices may even be detrimental and lead to higher subjective stress.^20^ This increased mental workload may in turn lead to worse performance.^21^ Eye-tracking technology may allow the assessment of visual attention (VA) of healthcare providers during resuscitation.^22,23^ This may allow quantification of distractions during resuscitation and in turn lead to strategies to overcome these during resuscitation.

We therefore aimed to study the impact of feedback devices on resuscitation quality with eye-tracking to analyze participants’ performance when supported with feedback devices.

## Methods

This study was a prospective randomized cross-over simulation-based trial conducted at the Medical University of Vienna, Austria. The study protocol was reviewed according to the Consolidated Standards of Reporting Trials (CONSORT).^24^ The ethics committee of the Medical University of Vienna gave this study an exempt status.

### Participants

Medical students in their final year, fellows, nurses, and consultants from our Neonatal Intensive Care Unit were eligible for enrolment.

### Study procedure and scenario

**Fig. 1:**
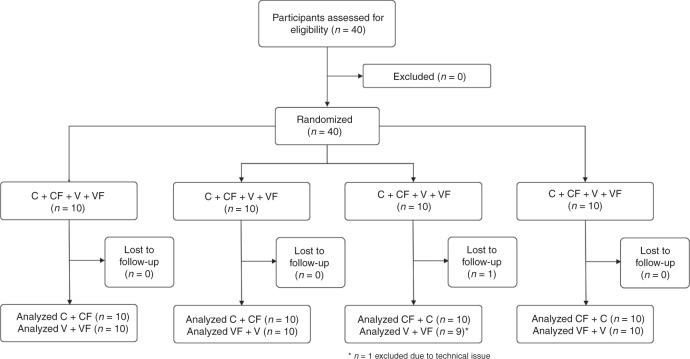
Flow diagram. C + CF chest compression without/with feedback device, V + VF ventilation without/with feedback device.

Participants received a brief review of the current pediatric CPR guidelines and an introduction to the feedback devices at the beginning of the study. Participants then signed an informed consent form and completed a questionnaire to assess demographic variables and expertise in pediatric resuscitation. Thereafter, eye-tracking glasses (Tobii 2.0, Tobii AB, Danderyd, Sweden) were calibrated as recommended by the company. Resuscitation teams consisted of the participant and a study nurse. Each participant completed a total of four basic life support scenarios according to the ERC pediatric BLS guidelines (15:2) in a cross-over setting.^25^ Each participant did CC twice, and ventilations (V) twice afterward (see Fig. 1).

### Randomization

The visibility of the feedback device was randomized (sealed envelope) within each pair of scenarios. Depending on group allocation (feedback condition vs. no-feedback condition), the feedback device was either visible or hidden from the participant, but always recording. Accordingly, the four scenarios were:

1. Participant performing chest compressions without feedback (=C).
2. Participant performing chest compressions with feedback (=CF).
3. Participant performing ventilations without feedback (=V).
4. Participant performing ventilations with feedback (=VF).

Each scenario lasted for 3 min and all tasks were done in one session. Immediately after each scenario, participants were asked to report perceived workload using the standardized NASA-Task Load Index (NASA-TLX).^26^ We measured the mark made by participants on the scale for the NASA-TLX score.

### Equipment

For CC scenarios we used the QCPR Baby manikin (Laerdal Medical, Stavanger, Norway), whereas for the ventilation scenarios we used the SimNewB manikin (Laerdal Medical, Stavanger, Norway) as it has no internal air leak. The QCPR Baby was connected to the SimPad Plus Skill Reporter device (Laerdal, Stavanger, Norway), which provided real-time visual feedback for CC performance. For ventilation feedback, a flow sensor (Neo Training, Monivent AB, Gothenburg, Sweden) was placed between the face mask (CareFusion Vital Signs Infant Face Mask, Chateaubriant, France) and the bag (Laerdal Silicone Resuscitator Pediatric Basic, Stavanger, Norway) to measure and show inspiratory (*V*_Ti_) and expiratory tidal volume (*V*_Te_, graphically and numerically), peak inspiratory pressure (numerically), and mask leak (leak, numerically) in real time. A tablet (iPad, Apple Inc., Cupertino, CA) recorded the parameters from the sensor wirelessly for further analysis. Participants wore Tobii 2.0 mobile eye-tracking glasses (Tobii, Danderyd, Sweden) to record real-time gaze behavior in all scenarios.

### Outcomes

The primary outcome was the quality of CCs and ventilations. The quality of CCs was measured with a total compression score (%), which is automatically calculated by the device including CC rate, CC rate compliance (percentage of correct CC rate), depth, depth compliance (percentage of correct CC depth), complete release, and hand position. Ventilation parameters included positive inspiratory pressure (<30 cmH_2_O),^27^ tidal volumes (4–8 mL/kg),^12,28^ and leakage (as low as possible). We evaluated whether the use of feedback devices improved or worsened the quality of CPR as compared to no-feedback conditions.

As secondary outcomes, we evaluated participants’ dwell time (amount of time looking at an area of interest [AOI]) and subjective workload. AOI describes a specific AOI in the video recording that is defined by researchers in the analysis process. For dwell time, the study group determined six AOIs: (1) feedback device (if available), (2) ventilation bag, (3) infant chest, (4) ventilation mask, (5) study nurse, and (6) others. Participants’ workload was measured with the NASA-TLX.^26^

### Sample size

The sample size calculation with the G*Power software for a paired *t* test with two repeated measures (feedback, no-feedback) revealed that a sample size of 34 participants would provide sufficient power (0.80) to detect an effect at the alpha level of 0.05 with medium effect size (*d*_*z*_ = 0.50).^29^

### Statistical analysis

Descriptive statistics were used to describe the sample and analyze the acceptance of feedback devices. Paired *t* tests were used to examine whether resuscitation quality, VA, and workload changed across the feedback conditions. Multiple regression analyses were conducted to test whether changes in resuscitation quality were predicted by participants’ expertise, VA, and workload. For *V*_Te_ and *V*_Ti_ scores, *χ*^2^ tests were applied to compare the proportion of scores within the 4–8 mL/kg range across the feedback conditions. All analyses were performed with SPSS 24.0 (IBM Corp., Armonk, NY). The level of significance was set at *P* < 0.05 (two-tailed). Cohen’s *d*_z_ (for paired *t* tests) were used as the measure of effect size, with 0.20, 0.50, and 0.80 indicating small, medium, and large effect, respectively. Parameters with a skewed data distribution were log transformed before analysis.

## Results

Data from 40 participants (25 females and 15 males) were collected. The recruitment period was from May 2019 to June 2020. Participants’ characteristics are shown in Table 1.

**Table 1:** Characteristics of study participants (*N* = 40).

Characteristic	*N* (%)	Mean (SD)	Range
Sex			
Female	25 (63)		
Male	15 (38)		
Age			
<30 years	19 (48)		
31–40 years	16 (40)		
41–50 years	4 (10)		
>51 years	1 (3)		
Occupation, level of training			
Neonatal nurse	1 (3)		
Medical student	9 (23)		
Neonatal fellow	22 (55)		
Neonatal consultant	8 (20)		
Clinical experience in neonatology (years)		4.26 (6.5)	0–26
Simulation-based resuscitation training (times)		5.34 (5.5)	0–30
Last CCs on actual patient			
Never	13 (33)		
<3 months	6 (15)		
3–5 months	5 (13)		
6–11 months	8 (20)		
>12 months	8 (20)		
Last CCs on manikin			
Never	0 (0)		
<3 months	18 (45)		
3–5 months	4 (10)		
6–11 months	8 (20)		
>12 months	10 (25)		
Perceived BLS competence			
Not sure	3 (8)		
Adequate	25 (64)		
Excellent	11 (28)		
Perceived ALS competence			
Not sure	19 (48)		
Adequate	16 (40)		
Excellent	5 (13)		
Experience with any feedback device before the study			
No	26 (65)		
Yes	14 (35)		

The clinical experience in neonatology and simulation-based resuscitation training variables were measured as continuous variables.

*CCs* chest compressions, *BLS* basic life support, *ALS* advanced life support.

### Resuscitation quality

#### CC quality

Participants showed significant improvements in several CC parameters when provided with real-time feedback (Table 2). In particular, they had a 22% higher total CC score and a 25% higher CC rate compliance than in the no-feedback condition (both *P* < 0.001). Self-reported CC quality increased by 7% (*P* = 0.03). Mean CC depth increased by 0.9 mm with feedback (*P* = 0.01), without having an effect on CC depth compliance. Years of clinical practice did not predict any changes in CC parameters, indicating that participants benefited from real-time feedback regardless of their experience.

**Table 2:** Differences in outcome variables between the “feedback” and “no-feedback” condition in the chest compression task.

Variable	No feedback (mean ± SD)	Feedback (mean ± SD)	Difference	*T* test^a^	
				*P*	*d*_z_
Chest compression (CC) quality					
Total CC score (%)	71.98 ± 31.4	93.95 ± 7.7	+21.97	**0.000**	0.91
Correct hand position (%)	82.48 ± 29.9	95.75 ± 7.8	+13.27	0.057	0.31
CC depth (mm)	40.85 ± 2.9	41.73 ± 1.6	+0.88	**0.013**	0.41
CC depth compliance (%)	85.90 ± 27.6	92.38 ± 14.3	+6.48	0.772	0.05
CC rate (/min)	120.23 ± 14.3	113.20 ± 8.9	−7.03	**0.000**	0.66
CC rate compliance (%)	47.03 ± 36.1	72.23 ± 28.1	+25.20	**0.000**	0.84
Full release (%)	82.23 ± 30.2	89.78 ± 17.3	+7.55	0.751	0.05
Self-reported CC quality (%)	64.19 ± 19.8	71.35 ± 13.1	+7.16	**0.031**	0.35
Visual attention (dwell time)					
Feedback device (%)	–	48.72 ± 23.8	–	–	–
Ventilation bag (%)	0.77 ± 2.3	0.21 ± 0.5	−0.56	0.092	0.28
Infant chest (%)	72.93 ± 24.1	32.64 ± 20.2	−40.29	**0.000**	1.73
Ventilation mask (%)	21.94 ± 22.9	12.74 ± 11.2	−9.20	**0.014**	0.41
Study nurse (%)	0.33 ± 0.6	0.22 ± 0.4	−0.11	0.264	0.18
Others (%)	4.02 ± 6.4	5.47 ± 8.9	+1.45	0.065	0.31
NASA TLX workload					
Average (%)	33.23 ± 14.1	36.82 ± 12.9	+3.59	**0.018**	0.39
Mental demand (%)	23.70 ± 19.7	31.93 ± 21.8	+8.23	**0.006**	0.46
Physical demand (%)	48.06 ± 21.2	46.79 ± 22.2	−1.72	0.572	0.09
Temporal demand (%)	30.24 ± 20.9	28.09 ± 18.7	−1.85	0.263	0.18
Performance demand (%)	34.08 ± 17.4	40.10 ± 15.7	+6.02	**0.047**	0.32
Effort (%)	46.23 ± 20.9	47.85 ± 21.5	+1.65	0.471	0.11
Frustration (%)	17.30 ± 16.5	26.09 ± 17.6	+8.79	**0.002**	0.54

Data are presented as mean ± SD. Positive difference values indicate an increase (and negative values a decrease) in the respective parameter from the no-feedback to feedback condition.

^a^Exact significances (*p* values) and effect sizes (Cohen’s *d*_z_ values) for paired *t* tests. Boldface *P* values indicate significant changes in the outcome variables between the feedback conditions.

#### Ventilation quality

Data of one participant were excluded because of technical issues with the flow sensor. Participants showed improvements in *V*_Ti_, *V*_Te_, and mask leak when provided with real-time feedback (Table 3). The mean *V*_Ti_ improved with feedback, increasing from 18% without feedback to 33% with feedback (*χ*^2^ = 6.27, *P* = 0.012). The mean *V*_Te_ was similar in both feedback conditions, but the proportion of *V*_Te_ in the 4–8 mL/kg compliance range increased from 36% in the no-feedback condition to 67% in the feedback condition (*χ*^2^ = 16.05, *P* < 0.001). Mask leak was 7.5% lower with feedback (*P* = 0.009). Participants with less experience were lower than a *V*_Te_ of 4–8 mL/kg limit more frequently than their more experienced counterparts (*β* = 0.45, *P* < 0.001).

**Table 3:** Differences in outcome variables between the “feedback” and “no-feedback” condition in the ventilation task.

Variable	No feedback (mean ± SD)	Feedback (mean ± SD)	Difference	*T* test^a^	
				*P*	*d*_z_
Ventilation quality					
Inspiratory tidal volume (mL/kg)	12.83 ± 6.0	10.15 ± 4.6	−1.68	**0.002**	0.54
Expiratory tidal volume (mL/kg)	7.34 ± 3.5	6.81 ± 2.6	−0.53	0.174	0.23
Peak inflation pressure (cm H_2_O)	23.90 ± 9.4	23.67 ± 8.5	−0.23	0.829	0.04
Mask leak (%)	31.76 ± 23.4	24.10 ± 18.6	−7.66	**0.009**	0.44
Self-reported quality (%)	51.93 ± 22.5	45.83 ± 29.0	−6.10	0.165	0.23
Visual attention (dwell time)					
Feedback device (%)	–	40.12 ± 18.8	–	–	–
Ventilation bag (%)	1.20 ± 2.4	0.84 ± 1.7	−0.36	0.272	0.18
Infant chest (%)	52.14 ± 24.5	23.43 ± 17.2	−28.71	**0.000**	1.21
Ventilation mask (%)	39.90 ± 22.5	29.05 ± 16.9	−10.85	**0.002**	0.53
Study nurse (%)	0.28 ± 0.6	0.11 ± 0.2	−0.17	0.055	0.32
Others (%)	6.83 ± 7.3	6.11 ± 5.7	−0.62	0.474	0.11
NASA TLX Workload					
Average (%)	28.14 ± 14.6	35.61 ± 16.0	+7.47	**0.000**	0.85
Mental demand (%)	29.03 ± 19.9	41.05 ± 23.8	+12.02	**0.000**	0.64
Physical demand (%)	23.03 ± 18.7	21.51 ± 18.5	−2.48	0.453	0.12
Temporal demand (%)	23.99 ± 16.9	23.38 ± 17.2	−0.61	0.613	0.08
Performance demand (%)	39.56 ± 18.5	50.32 ± 23.2	+10.76	**0.002**	0.53
Effort (%)	28.90 ± 22.2	33.73 ± 25.2	+4.83	0.078	0.29
Frustration (%)	24.58 ± 19.7	43.78 ± 28.9	+19.20	**0.000**	0.73

Data are presented as mean ± SD. Positive difference values indicate an increase (and negative values a decrease) in the respective parameter from the no-feedback to feedback condition

^a^Exact significances (*P* values) and effect sizes (Cohen’s *d*_z_ values) for paired *t* tests. Boldface *P* values indicate significant changes in the outcome variables between the feedback conditions.

### Visual attention

#### VA in the CC task

In the no-feedback condition, infant chest was the most frequently attended AOI (73%), followed by ventilation mask (22%). The addition of a feedback device had a powerful effect on participants’ VA, with the feedback device being now the most frequently attended AOI (49%), followed by infant chest (33%) and ventilation mask (13%). Paired comparisons revealed that participants significantly reduced their attention to the infant chest and the mask in the feedback condition compared with the no-feedback condition (Table 2). Years of clinical practice did not predict changes in VA except for the feedback device AOI; more experienced participants tended to dwell on the device longer than less experienced participants (*β* = 0.34, *P* = 0.04). Furthermore, participants who dwelled longer on the device significantly reduced their mean CC rate (*β* = −0.29, *P* = 0.01), thereby exceeding the 120 CCs per minute limit less frequently and thus improving their CC rate compliance (113.2 with feedback vs. 120.2 without feedback).

#### VA in the ventilation task

Similar to the CC scenario, the infant’s chest was the most frequently attended AOI (52%), and the ventilation mask the second (40%) in the no-feedback condition. In the feedback condition, the feedback device was the most frequently attended AOI (40%), whereas participants’ attention to the infant’s chest and the mask declined significantly (Table 3). Years of clinical practice did not predict changes in VA except for the mask AOI; more experienced participants tended to dwell on the ventilation mask shorter than less experienced participants when provided with real-time feedback (*β* = −0.31, *P* = 0.03). Dwell time on the feedback device predicted changes in the mean *V*_Te_ (*β* = 0.23, *P* = 0.03), with participants who dwelled longer on the device having higher mean *V*_Te_ (7.0 mL/kg) than participants who attended to the device for a shorter time (6.6 mL/kg).

### Workload

#### Workload in the CC task

The average NASA workload in the feedback condition was 37%, which was ∼3.5% higher than in the no-feedback condition (*P* = 0.02). This was especially due to the increases in mental demands (*P* = 0.006), performance demands (*P* = 0.05), and frustration (*P* = 0.002) (Table 2). More experienced participants experienced higher average workload (*β* = 0.37, *P* = 0.001), higher mental demands (*β* = 0.39, *P* = 0.003), and put more effort into the task (*β* = 0.26, *P* = 0.02) than less experienced participants. The average workload in the feedback condition did not predict changes in participants’ actual CC quality. There were only two effects of the NASA subscales on self-reported CC quality: higher performance demand and higher frustration were related to lower self-reported CC quality in the feedback condition (*β* = −0.44, *P* = 0.003, and *β* = −0.50, *P* = 0.001, respectively).

#### Workload in the ventilation task

The average NASA workload in the feedback condition was 36%, which was ∼8% higher than in the no-feedback condition (*P* < 0.001). This was especially due to increases in mental demands (*P* < 0.001), performance demands (*P* = 0.002), and frustration (*P* < 0.001) (Table 3). The increase in workload was unrelated to years of clinical practice. The average workload, performance demand, and frustration were related to a decline in self-reported ventilation quality (*β* = −0.58, *P* < 0.001, *β* = −0.63, *P* < 0.001, and *β* =  −0.63, *P* < 0.001, respectively). In addition, higher frustration was related to higher *V*_Ti_ (β = 0.28, *P* = 0.04), with participants with *V*_Ti_ in the 4–8 mL/kg range being less frustrated than participants with *V*_Ti_ out of that range (28 vs. 53%).

### The acceptance of feedback device

The majority of participants found the use of both feedback devices, SimPad and Monivent, helpful for visualization of the CC and ventilation quality, respectively. More than 90% of participants perceived the feedback from the SimPad device as easily interpretable. The answers to the Monivent device were more ambiguous, with 62% of participants rating the feedback as rather easy to interpret and 36% of participants perceiving it rather difficult. Participants further stated that using feedback devices would also be beneficial in an actual scenario in a pediatric patient (Table 4).

**Table 4:** Participants’ subjective experience with the SimPad (chest compressions) and Monivent Neo (ventilation) feedback device.

	SimPad, *N* (%)	Monivent Neo, *N* (%)
Did you find the feedback device helpful in performing chest compressions/ventilations?		
Very helpful	20 (50)	21 (54)
Somewhat helpful	15 (38)	12 (31)
Moderate	2 (5)	1 (3)
Slightly helpful	2 (5)	4 (10)
Not at all helpful	1 (3)	1 (3)
How difficult or easy was it to interpret feedback from the device?		
Very difficult	0 (0)	0 (0)
Difficult	0 (0)	14 (36)
Moderate	3 (8)	1 (3)
Easy	22 (55)	15 (39)
Very easy	15 (38)	9 (23)
Do you think using a feedback device for chest compressions/ventilations would be beneficial in a real scenario?		
Yes	35 (88)	28 (72)
No	2 (5)	4 (10)
Don’t know	3 (8)	7 (18)
Did you get distracted by the feedback devices?		
Very distracted	5 (13)	
Somewhat distracted	21 (53)	
Moderate	5 (13)	
Slightly distracted	6 (15)	
Not at all distracted	3 (8)	

## Discussion

**Fig. 2:**
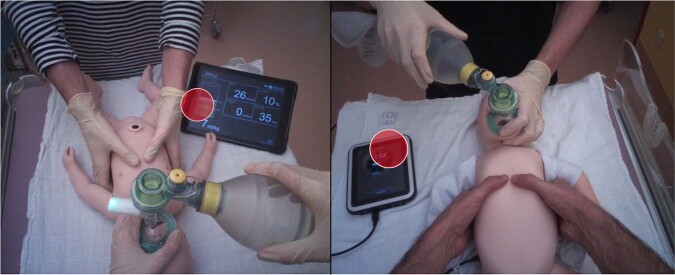
Snapshots showing visual attention (red circle) of participants when feedback devices were used. Participants’ attention shifted significantly to the device and away from the manikin. Dwell time (=total duration of visit time) on the feedback device was 40.1% in the ventilation feedback condition (left) and 48.7% in the chest compression feedback condition (right). In both conditions, participants significantly reduced attention from the infant’s chest and mask (72.9 vs. 32.6% and 21.9 vs. 12.7%).

In this study, we observed the effect of feedback devices on the quality of pediatric resuscitation with eye tracking to analyze participants’ attention and workload. There were three main findings: (i) As in previous studies, we found that performance quality improved with real-time feedback for both CCs and ventilations. Feedback devices are designed to assist in delivering optimal resuscitation quality.^6–10,12–15^ A recently published systematic review also supported the use of feedback devices for pediatric as well as adult resuscitation support.^30^ The feedback devices helped the practitioners to appropriately recognize and optimize resuscitative tasks rather than serving as a distraction.

(ii) The addition of a feedback device influenced VA. When feedback devices were used, participants’ attention shifted significantly to the device (see Fig. 2) and away from the manikin consistent with former studies.^31,32^ However, this shift in attention was not harmful to performance in our study. We further observed that more experienced participants spent more time looking at the feedback device than on the manikin. These participants may have needed more time to interpret the feedback from the device or relied on their haptic memory requiring less confirmation of their task by looking directly at the manikin. However, also the non-feedback group’s dwell time was mostly on the infant’s chest and mask, likely to determine adequate chest rise. Probably, the participants were looking for feedback or needed feedback on how they were doing by paying attention to the areas that would tell them the quality of ventilation. While it sounds reasonable that participants are actively seeking feedback on performance, there is no literature that confirms this so far.

(iii) The addition of a feedback device increased subjective workload, but this increase was small^33^ and did not interfere with resuscitation quality. The increase was markedly higher when the participant had more years of practice, but only when performing CCs. Interestingly, a higher subjective workload was commonly paired with frustration when using feedback devices, but the quality of CCs and ventilation did not suffer in our study. The relationship between workload and clinical performance has been reported controversially in the literature. Previous studies have described a potential reduction of workload when using feedback devices,^34^ but also an increased workload with improved quality of CC.^20^ While feedback devices have beneficial effects on resuscitation performance, the effect might decrease once feedback devices are used on a daily basis and are not special to providers anymore.^35^ Ultimately, these devices make CPR performance measurably and their clinical use may allow the identification of the best practice during resuscitation.^11^

Overall, our findings support previous reports that feedback devices may be an important tool in CPR in situ as well as in real life,^36^ also with an improvement of return of spontaneous circulation rates.^37^

However, while we are not able to provide clinical data, it was interesting to see that while feedback devices increased subjective workload, resuscitation quality was mostly not influenced. Future studies should also focus on interviews with participants after the scenario, to identify why and what exactly influenced workload. Most providers were using feedback devices for the first time, which might in part explain the higher perceived workload. It is also reasonable that repetitive and regular training with feedback devices might decrease subjective workload while still impacting the quality of resuscitation positively. Alternatively, this increase in workload with feedback devices may be offset by a resuscitation coach who relays CPR quality information to the resuscitating provider.^38,39^ We assume that adding live feedback devices in real clinical situations, such as cardiopulmonary resuscitation and positive pressure ventilation, can help to increase the quality of patient care. However, clinical studies analyzing the utility of feedback devices in such situations including VA and workload are needed.

### Limitations

This was a simulation-based trial not involving any real patients. Furthermore, the study design did not allow for blinding. Participants knew they were being studied, which might amplify the shift of VA to the feedback device. In addition, we were not able to evaluate any other feedback routes such as voice or sound cues. Furthermore, scenarios were limited to 3 min, so we were unable to identify possible changes of workload with a longer duration of resuscitation. Workload measurement was based on subjective reports by participants.

## Conclusion

We found that resuscitation quality significantly improved when using feedback devices in a simulated setting. VA shifted to the feedback device, when provided, and away from the simulated patient and other equipment. The subjective workload was higher with the feedback device. High-quality randomized controlled trials, as well as real-patient trials focusing on patient outcomes, are needed to determine the impact of VA and stressors when using feedback devices.

## Supplementary Information

Supplementary Information
